# Illegal Dumping of Toxic Waste and Its Effect on Human Health in Campania, Italy

**DOI:** 10.3390/ijerph120606818

**Published:** 2015-06-16

**Authors:** Alfredo Mazza, Prisco Piscitelli, Cosimo Neglia, Giulia Della Rosa, Leopoldo Iannuzzi

**Affiliations:** 1 General Hospital Sarno, Local Health Authority ASL Salerno, 84087 Sarno, Italy; 2 National Research Council (CNR), ISPAAM, 80100 Naples, Italy; E-Mail: leopoldo.iannuzzi@ispaam.cnr.it; 3 IOS, Southern Italy Hospital Institute, 80100 Naples, Italy; E-Mail: prisco.piscitelli@tiscali.it; 4 Coleman Ltd., 80100 Naples, Italy; 5 Euro Mediterranean Scientific Biomedical Institute, 72100 Brindisi, Italy; E-Mails: neglia@isbem.it (C.N.); dellarosa.giulia@gmail.com (G.D.R.)

**Keywords:** toxic waste, environmental pollution, public health, cancer mortality, waste emergency, Campania

## Abstract

The region of Campania (particularly Naples and Caserta) has experienced an emergency in the waste management cycle during past years. Although the most critical phase has been overcome after the construction of the incineration plant in Acerra (an old-fashioned technology built up over a few months, whose impact on environment and health has not yet been assessed), most of the underlying problems have not been resolved. The illegal burning of wheels, plastics, textiles, and other industrial residuals, along with the detection of two thousand toxic substance dumping sites, still represents major concerns of environmental pollution and population health. This review summarizes the most relevant studies, which analyzed chemical contamination (primarily dioxins and polychlorinated biphenyls (PCBs)) of the air, soil, water, animals, and humans in Campania. In addition, we reviewed information on population health (*i.e*., mortality data, congenital malformations, and cancer incidence). Moving from a detailed mapping of (mostly illegal) waste dumping sites in Campania, we have focused on recent studies which have found: (a) high concentrations of dioxins (≥5.0 pg TEQ/g fat) in milk samples from sheep, cows, and river buffaloes; (b) remarkable contamination of dioxin and PCBs in human milk samples from those living in the Naples and Caserta areas (PCDDs+PCDFs and dioxin-like-PCBs (dl-PCBs) assessed at 16.6 pg TEQ/g of fat; range: 7.5–43 pg/g of fat); (c) potential age-adjusted standardized mortality rates associated with some specific cancer types; (d) a statistically significant association between exposure to illegal toxic waste dumping sites and cancer mortality, even after adjustment by socio-economic factors and other environmental indicators.

## 1. Introduction

Several epidemiological studies have been recently performed in different countries about the putative adverse health effects associated with residence in the vicinity of toxic waste dump sites [1,2,3,4]. Although an increased risk of health concerns (especially for cancer and congenital malformations) has been reported, no causal association between pollution and diseases has been adequately confirmed. In this study, we have reviewed recent data about distribution of illegal waste dumping sites, as well as dioxin contamination of air, soil, water, animals, and humans in the Campania region. At the same time, we have examined available information on population health of those living in contaminated areas, especially Naples and Caserta (including mortality rates, congenital malformations, and cancer incidence).

During past decades, an illegal practice of industrial toxic and solid urban waste dumping occurred in the Campania region and Southern Italy [5]. Furthermore, tons of waste have been dumped in agricultural areas and illegally burned, usually during the night, releasing a number of dangerous chemicals, including dioxins, a large family of chlorinate compounds with 17 highly toxic molecules, including the 2,3,7,8-tetraclhorodibenzo-*p*-dioxins (TCDD), which has been recently classified as carcinogenic in both animals and humans by the International Agency for Research on Cancer (IARC) [6]. Although the emergency phase has been overcome after the construction of the incineration plant in Acerra, most of the underlying problems have not been resolved. The illegal burning of wheels, plastics, and textile or other industrial residuals, together with the detection of two-thousands sites of toxic substances dumping sites still represent major concerns for environmental pollution and population health [5].

Recent studies have provided evidence of an increased risk of liver and lung cancer, as well as lymphopoietic neoplasms associated with 2,3,7,8-TCDD [7,8,9], despite the fact that a correlation of such specific diseases with individuals overexposed to 2,3,7,8-TCDD or to dioxins in general is still controversial and yet to be demonstrated [10,11,12,13,14]. In addition, sheep that are bred within contaminated areas and exposed to dioxins have been demonstrated to present higher rates of chromosome fragility, higher mortality, and a higher incidence of abnormal fetal development when compared with sheep raised in non-contaminated areas [15]. Assessment of dioxins in milk samples from sheep, buffalo, and cattle raised in the Campania region revealed that about one fourth of them presented levels of dioxins much higher than the normal threshold indicated by European Commission (3.0 pg TEQ/g fat) [16,17]. Actually, according to the latest EU-regulation 1259/2011, the new limit for polychlorobenzodioxins (PCDDs) and polychlorodibenzofurans (PCDFs) in the milk has been lowered to 2.5 pg TEQ/g of fat and the limit of PCDDs+PCDFs+dlPCBs sum is 5.5 pg TEQ/g of fat).

Cancer mortality and congenital malformations have been reported in some areas of the Campania region under intense environmental pressure [18]. A recent review by Triassi *et al*., taking into account all available findings [19], underlined the possible long-term role of waste and its positive correlation with outcomes such as liver and lung cancer and mortality in humans living in the Caserta and Naples provinces, in addition to a short-term (less than one year) waste-related effect, confirmed by the association with congenital malformation already documented [18]. However, the authors concluded that further studies are needed to better define waste-related health effects, since updated data are still far from being conclusive [19].

In a previous study, we have reported a higher standardized mortality rate in a restricted area of the Campania region, known as “The Italian triangle of death,” compared to both national and regional average values [20]. The same conclusion was recently reported by the Italian Institute of Health (ISS) [21]. In this study, we have reviewed recent data about the distribution of illegal waste dumping sites, environmental pollution, and animal and human contamination with dioxins, polychlorinated biphenyls (PCBs), and other toxic chemical compounds, in addition to the most relevant available information on population health (including overall and cancer-specific mortality) in the Campania region.

## 2. Review Methodology

We have reviewed available evidence published in international medical literature and technical reports issued by local, regional, national, and international environmental agencies, as well as healthcare and food safety authorities, concerning human exposure to pollutants and carcinogenic substances in the Campania region. We have carried out an updated systematic review on both *PubMed* and *Embase* databases, seeking research in medical literature published up to December 2014, which addressed the issue of illegal dumping of toxic waste and human health in Campania (inclusion criteria). The methodological quality of studies addressing the issue of early osteoarthritis was evaluated according to the Levels of Evidence defined by the Centre for Evidence Based Medicine (CEBM). Articles written in languages other than English (*i.e.*, Italian) were included if they could add any relevant information to the current knowledge about our topic of study. Official reports issued by different health or environmental authorities such as the ISS and the National Institute for Environmental Protection (ISPRA), food safety research/control institutions (*i.e.*, Zooprophylactic Institute of Teramo), the National Institute for Statistics (ISTAT), and the Campania Region Environmental Protection Agency (ARPAC) have been included in our search. Interpretation of the findings has been conducted in the frame of current medical knowledge.

## 3. Results and Discussion

### 3.1. Waste Dumping Sites in the Campania Region

**Figure 1:**
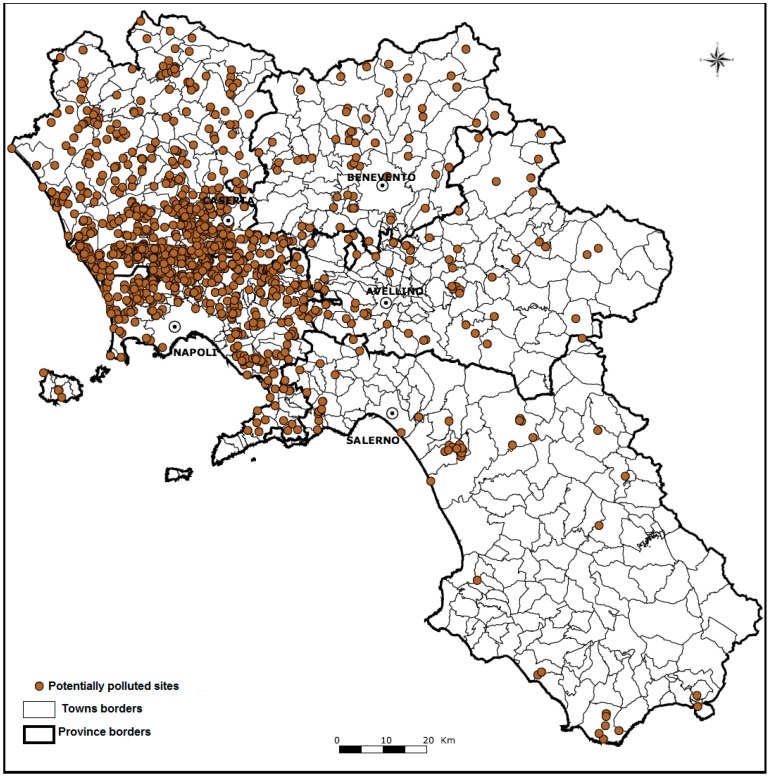
Map of illegal toxic waste dumping sites in the region of Campania, Italy (Official Datasource: ARPAC) [22].

In Figure 1, the geographical distribution of illegal waste dumping sites identified in the Campania region, according to investigations carried out by ARPAC [22], is shown. The polluted sites are mostly concentrated in the north-western area of the Campania region (corresponding to the provinces of Naples and Caserta). The epidemiological study by ARPAC, as well as reports from other authors, revealed an excess of risks for human health among individuals living in this geographical area. In particular, both risks for cancer mortality (all types of malignancies) and, to a less extent, for congenital malformations (again, in all anatomical sites) have been found much higher than expected [8,9,18,19]. However, any association between the illegal practice of dumping toxic waste and adverse effects on human health should be considered with caution since all these studies did not take into account the coexistence of possible additional risk factors (such as smoking habits, lifestyle, occupation, *etc*.). Nevertheless, the knowledge of disease distribution in such a geographical area could be really helpful to draw a map of risks, as the first step toward further epidemiological, clinical, and toxicological investigations, in order to more appropriately assess the causal relations.

### 3.2. Dioxins in Milk Specimens within the Provinces of Naples and Caserta

Dioxins do accumulate within the food chain due to their high lipophilicity and stability [23]. Food consumption is the most important source of dioxin exposure in humans. In Europe and America, dioxin exposure is primarily due to the consumption of animal products (meat, eggs, and dairy) [24,25]. Evaluation of the dioxin concentration in milk samples from mammals bred in a specific geographical area could represent a quite reliable index of background levels of dioxin contamination within that territory. During past years, several analyses have been carried out for detection of dioxins in mammalian milk produced by domestic animals (sheep and cows) from several farms located in the same north-western area of the Campania region [16,26,27]. Among tested samples, about two thirds of them presented a level of dioxins exceeding 3.0 pg TEQ/g fat, which represents the safety threshold indicated by the European Commission before 2011, and about one fourth of them presented a concentration higher than 5.0 pg TEQ/g fat [25,26] (*actually the permitted values of PCDDs and PCDFs has been reduced to 2.5 pgTEQ/g of fat and the sum of PCDDs+PCDFs+dlPCBs to 5.5 pgTEQ/g of fat*). Moreover, among the 17 different types of dioxins found in the milk mass, 2,3,7,8-TCDD showed a mean value of 1.44 pg TEQ/g fat in 90% of the tested samples [24,25]; this concentration is remarkably higher (about 13 times) than the mean value of 0.11 pg TEQ/g fat, which usually reported in Italy [26]. The highest values of 2,3,7,8-TCDD were observed in milk samples from sheep bred in the province of Naples [16,27]. Finally, a contamination of PCBs in milk samples produced by sheep, cattle, and buffaloes raised within the same area of Campania has been reported [27]. In particular, about one fourth of milk samples presented a level of PCBs higher than 5.0 pg TEQ/g fat [26,27]. This latter finding confirms that PCBs continue to be heavily diffused into the environment [26]. Since humans are at the top of the food chain, with the highest capability to concentrate dioxins in their fat tissues, continuous monitoring of dioxin levels in humans, who live in hypothetically contaminated geographical areas (such as Campania), is also required to gauge exposure.

### 3.3. Dioxins and PCBs Concentrations in Human Milk Samples from the Naples and Caserta Areas

The issue of human contamination with dioxins (PCDDs, PCDFs) and dioxin-like-PCBs (dl-PCBs) has been specifically assessed in recently published studies carried out at a time when no legal waste incineration plants in Campania were present [28]. This study analyzed concentrations of PCDD, PCDFs and dl-PCBs in individual milk samples of 94 young breastfeeding women (aged 19–32) living in the Naples and Caserta provinces according to the WHO standardized protocol, finding out that: (1) all milk samples were contaminated by dioxins and dl-PCBs with an average level of dioxins of 16.6 pg TEQ/g of fat (ranging from 7.5 to 43 pg TEQ/g of fat); (2) their concentrations significantly increased with age (*p* < 0.01). It is well known that dioxin and dl-PCBs concentrations significantly increased with age in all areas considered, including those in this study. This information clearly indicated that the average contamination of the study area of Caserta and Naples is similar to that of the cities of Milan and Piacenza, but the highly contaminated municipalities of Caserta and Naples have dioxin concentrations that are up to 1.5 times the maximum contamination recorded in Milan by Ulaszewska *et al*. [29]. However, we found a lower concentration of dioxins and dl-PCBs with respect to studies carried out in Duisburg (Germany) on individual samples. In a separate paper concerning the same population study, a significant increase in dioxin concentration in those subjects with more exposure to the burning of waste (*p* < 0.05) was confirmed by using, as a measure of environmental risk of dioxins (EDR), the values of dioxin concentration in buffalo milk samples collected in the study area [30]. In this latter study, dioxin levels in 94 individual breast milk samples were significantly correlated to the EDR, the age of the sampled women, and the presence of the illegal burning of solid waste [30].

### 3.4. Overall Mortality in Campania, and Cancer-Specific Mortality in the Provinces of Naples and Caserta

According to the national statistics, mortality rates per 100,000 inhabitants in the Campania region are remarkably higher than average Italian values for “all causes” of death both in men and women (Table 1). Furthermore, historical mortality data show that overall cancer mortality rates of the Campania region in the 1990s were lower than the Italian average values, while they are currently higher than the national rates (Table 1) [31]. Despite that, a decreasing trend both in general mortality for all causes and overall cancer mortality has been observed from 1990 to 2012.

The standardized mortality ratio (SMR) has been calculated in the five provinces of the Campania region (including Salerno, Avellino, and Benevento, in addition to Naples and Caserta) for the period 1998 to 2001 [7]. The age-adjusted SMRs for all causes, for all cancers, and for cardiovascular diseases are significantly higher than expected into the provinces of Naples and Caserta, whereas they are lower than expected in the remaining three provinces of Salerno, Avellino and Benevento (Table 2). The SMR for each province was defined as the ratio of observed deaths in that province, as registered by the local health authorities (ASL), compared to the expected amount of deaths in a control population from the same Campania region. The provinces of Caserta and Naples, which include the above-mentioned critical area, presented the highest and most significant SMR values for stomach and digestive system cancers, liver carcinoma, and cancer of the pleura (Table 2). Data provided by the only local cancer registry (which covers only a part of the Naples province) concerning cancer incidence trends from the end of the 1990s to 2012 have shown increases in breast and lung cancer in women and lung, prostate, and colorectal cancer in men, with peaks in 2012 observed for breast and colorectal cancer (34,000 and 22,000 prevalent cases in women and men, respectively). The highest mortality rate was recorded in 2012 for lung cancer in men and breast cancer in women (80 and 31 per 100,000, respectively) [32].

**Table 1:** Mortality rates per 100,000 inhabitants for all causes of death and for overall cancers from 1990 to 2012 in the Campania region *vs.* Italy (data from 2004 and 2005 are not available), as provided by the Italian National Institute for Statistics (ISTAT) and Italian Institute of Health (ISS). [30].

Year	Men				Women				Total (Men + Women)			
	Std Rate Campania All Causes	Std Rate ITALY All Causes	Std Rate Campania Overall Cancer	Std Rate ITALY Overall Cancer	Std Rate Campania All Causes	Std Rate ITALY All Causes	Std Rate Campania Overall Cancer	Std Rate ITALY Overall Cancer	Std Rate Campania All Causes	Std Rate ITALY All Causes	Std Rate Campania Overall Cancer	Std Rate ITALY Overall Cancer
**1990**	663.7	*626*.*7*	174	*192*.*7*	422.3	*356.7*	90*.*1	*100.5*	543	*491.7*	132.1	*146.6*
**1991**	687.0	*623.2*	179.5	*190.8*	422.9	*352.5*	93.7	*100.4*	554.9	*487.8*	136.6	*145.6*
**1992**	662.6	*599.8*	178.6	*187.3*	403.6	*339.3*	91.3	*98.5*	533.1	*469.5*	134.9	*142.9*
**1993**	642.2	*587.2*	177.2	*186.2*	401.0	*335.8*	90.8	*99*	521.6	*461.5*	134.0	*142.6*
**1994**	642.2	*578.8*	180.9	*185.7*	393.5	*330.0*	95.5	*98.5*	517.8	*454.4*	138.2	*142.1*
**1995**	617.4	*564.6*	173.1	*178.4*	366.9	*315.7*	90.9	*94.4*	492.1	*440.2*	132.0	*136.4*
**1996**	602.9	*547.8*	173.3	*177.2*	354.3	*306.3*	89.4	*94.1*	478.6	*427.1*	131.4	*135.6*
**1997**	602.9	*536.1*	174.6	*174.3*	346.4	*300.3*	86.9	*91.6*	474.7	*418.2*	130.8	*132.9*
**1998**	596.7	*527.9*	176.4	*171.8*	346.8	*297.0*	89.4	*90.6*	471.8	*412.4*	132.9	*131.2*
**1999**	572.7	*506.7*	171.1	*166.7*	334.4	*285.3*	84.4	*87.553*	439.5	*382.2*	121.6	*121.3*
**2000**	561.7	*488.3*	170.1	*163.7*	336.7	*278.5*	89.5	*89.653*	435.7	*370.2*	123.9	*121.1*
**2001**	535.6	*476.7*	167.9	*164.2*	322.0	*271.8*	88.7	*89.226*	415.7	*361.7*	122.4	*121.2*
**2002**	530.1	*467.6*	168.3	*159.8*	316.7	*269.2*	85.7	*88.343*	411.5	*356.4*	121.4	*118.8*
**2003**	532.7	*471.7*	166.8	*159.0*	321.1	*277.7*	86.6	*88.654*	415.0	*363.0*	120.9	*118.6*
**2006**	471.2	*416.8*	157.5	*149.2*	282.6	*243.8*	84.8	*84.162*	366.6	*320.4*	116.1	*111.9*
**2007**	473.5	*408.7*	159.1	*146.2*	288.2	*243.1*	86.6	*84.66*	371.7	*316.5*	118.2	*110.9*
**2008**	457.2	*400.2*	155.2	*143.1*	277.0	*239.1*	83.9	*83.659*	357.7	*310.7*	114.9	*109.1*
**2009**	451.1	*392.5*	151.9	*140.7*	278.3	*237.6*	83,6	*83.639*	355.9	*306.5*	113.4	*108.1*
**2010**	440.3	*378.0*	153.5	*137.4*	270.7	*228.1*	85.0	*82.019*	347.1	*294.7*	114.9	*105.7*
**2011**	437.6	*375.7*	150.5	*136.1*	273.6	*229.3*	85.3	*81.937*	347.0	*294.5*	113.5	*105.2*
2012	434.8	*376.0*	148.8	*134.7*	272.2	*232.4*	85.2	*82.108*	345.1	*296.4*	112.8	*104.7*

**Table 2:** Age-adjusted standardized mortality ratios (SMRs) by causes of death for the period of 1998 to 2001 in males (M) and females (F) of five provinces of the Campania region. Data are elaborated from “Atlas of Mortality in Campania Region 2005” [7].

Causes of Death (ICD-IX Codes)	Avellino		Benevento		Caserta		Naples		Salerno	
	M	F	M	F	M	F	M	F	M	F
All causes (001–999)	83.6	86.3	84.9	82.4	104.9 *	106.1 *	109.3 *	108.3 *	91.0	90.8
All cancers (140–208)	80.4	77.1	81.9	84.1	104.5 *	100.7	111.7 *	109.8 *	87.1	92.2
Esophagus (150)	84.2	41.9	166.5 *	35.3	86.7	90.7	110.2	130.3 *	72.4	89.3
Stomach (151)	102.4	91.0	86.9	107.4	135.5 *	122.9 *	102.6	101.0	75.0	84.6
Colon (153)	96.3	66.6	106.4	104.9	106.6	110.7	106.2	107.7	84.5	89.7
Rectum (154)	101.8	70.7	87.7	86.9	109.3	102.4	105.8	106.0	85.5	102.9
Liver (155)	63.4	60.3	59.1	53.8	97.7	100.8	123.9 *	120.8 *	79.5	85.7
Pancreas (157)	86.3	83.0	94.9	84.6	96.3	92.8	107.3	110.5 *	94.4	93.5
Other digestive (159)	51.7	67.5	60.7	83.3	126.8 *	127.6 *	114.1 *	103.7	91.4	95.2
Lung (162)	70.2	61.0	72.2	65.0	105.0	82.0	116.6 *	128.6 *	83.2	73.5
Pleura (163)	45.3	116.7	48.9	15.0	49.9	68.4	134.0 *	138.6 *	96.8	46.8
Breast (174)	−	68.7	−	84.4	−	104.2	−	110.2	−	91.2
Prostate (185)	89.5	−	87.5		103.7		107.7 *		93.3	
Bladder (188)	83.7	66.6	71.7	84.8	96.2	93.2	114.6 *	117.7 *	90.6	87.2
Kidney (189)	91.1	70.4	88.1	86.5	102.0	80.0	102.1	127.9 *	101.8	66.7
Brain (191)	73.4	59.0	93.4	99.4	113.6	95.8	105.6	100.2	90.9	120.3
Thyroid (193)	103.3	94.3	55.9	91.6	110.0	86.7	101.3	107.3	102.3	97.2
Hodgkin’s and non Hodgkin’s lymphoma (200, 202)	88.0	102.7	111.1	102.8	90.6	84.5	100.8	108.3	106.5	88.7
Multiple Myeloma (203)	111.7	118.7	82.1	72.9	97.1	63.8	97.6	105.8	107.4	110.6
Leukemia (204–208)	113.9	79.10	98.1	110.2	111.4	99.9	100.4	105.6	85.9	93.0
Diabetes mellitus (250)	73.7	70.2	68.8	61.8	100.7	107.7	110.4 *	115.2 *	101.3	89.5
All cardiovascular diseases (390–459)	85.9	92.0	89.1	86.2	107.4 *	110.5 *	107.6 *	106.1 *	91.8	89.9
Congenital malformations (740–759)	67.9	90.8	109.7	115.7	115.1	87.7	101.9	102.2	90.9	103.3

* Statistically significant (*p* < 0.05).

### 3.5. Association between Waste Pollution and Cancer Mortality

The relative risk estimates of cancer mortality have been associated with exposure to toxic and solid wastes within the provinces of Naples and Caserta. In particular, the Italian Civil Protection Department conducted an epidemiological correlation study to estimate the association between exposure to illegal waste dumping and adverse health effects in areas encountering this environmental problem, adjusted by several socioeconomic factors [33]. The exposure to waste was measured as the Environmental Waste Index (EWI). The EWI is the result of a complex statistical analysis that accounts for population density, distance to a waste site, volume and type of waste, and routes of contamination (e.g., food, air, or water). The adjustment by the socioeconomic factors was performed by including a variable called the index of socioeconomic deprivation (ID), which accounts for variables such as education, income, and employment, into a regression model. [34]. This study was conducted in 196 municipalities, during the period of 1994 to 2001; among them, 92 were located in the province of Naples (including the city of Naples) and 104 were located in the province of Caserta [33]. These municipalities were grouped into five classes according to the EWI, where class I has the lowest EWI and class V has the highest EWI; statistical analyses were conducted using a Poisson regression model. The authors observed that the relative risks of mortality (for all causes) as well as of the overall cancer mortality were significantly associated with the increase of EWIs, adjusted by environmental and socioeconomic indicators [32]. Results are summarized in Table 3. The highest increase in mortality risk was observed for liver and biliary tract cancers in both males and females (Table 3). This study also found a statistically significant association between EWI and congenital malformations of the nervous and urogenital systems (Table 3). Compared to the municipalities with lowest EWI (class I), the municipalities with the highest EWI (class V) presented an 83% increase in the risk of congenital malformations of the nervous and urogenital systems (Table 4). It is worth underlining that the municipalities with the highest EWI were located into the north-western area of the Campania region, where most illegal waste dumping sites have been identified (Figure 1).

**Table 3:** Estimated relative risks (RR) of cause-specific mortality in 196 municipalities in the provinces of Naples and Caserta for the period of 1994 to 2001. The estimated RR is adjusted for socioeconomic factors. The last column (Trend) summarizes the percent increase in mortality associated with a one-unit increase in the EWI class. Data are elaborated from the Italian Government (Department of Civil Protection). [32]

Cause of Death	Class of Environmental Waste Index (EWI)					
	I	II	III	IV	V	Trend
All causes mortality	1/1	1.05 */1.02	1.08 */1.08 *	1.04 */1.05 *	1.09 */1.12 *	1.02 */1.02 *
All cancers	1/1	1.04 */1.05 *	1.06 */1.02	1.05 */1.04	1.04/1.07 *	1.01 */1.01 *
Lung cancer	1/1	1.05 */1.45 *	1.06/1.14	1.06/1.06	1.07/1.09	1.02 */0.98
Liver and biliary tract cancer	1/1	0.91/0.91	1.21 */1.09	1.01/1.10	1.19 */1.29 *	1.04 */1.07 *
Stomach cancer	1/1	1.03/0.92	1.03/0.94	1.19 */1.02	1.16/1.17	1.05 */1.03
Bladder cancer	1/1	1.12/1.08	0.94/0.87	1.07/0.97	0.96/0.83	0.99/0.97
Kidney cancer	1/1	0.97/1.07	0.99/1.11	0.85/1.03	0.83/1.19	0.96/1.02
Soft tissue cancer	1/1	0.90/1.08	0.80/1.84	0.69/1.34	1.25/1.00	0.96/1.08
Non-Hodgkin Lymphoma	1/1	1.09/1.10	1.25/1.04	1.07/1.20	0.96/1.00	1.01/1.02

* Statistically significant (*p* < 0.05).

### 3.6. Discussion

This study summarizes the most recent and valuable scientific papers about environmental pollution due to illegal dumping or burning of waste (both urban and toxic ones) and animal or human contamination in the Campania region. Consequently, the main limitation of our work consists in the few numbers of studies available on the specific topic addressed, also taking into account that environmental exposure-related diseases require a long time before a diagnosis occurs.

**Table 4:** Estimated relative risks (RR) of congenital malformations in 196 municipalities in the provinces of Naples and Caserta for the period of 1994 to 2001. The RR is adjusted for environmental and socioeconomic indicators. The last column (Trend) summarizes the percent increase in the health outcome associated with a one-unit increase in the EWI class. Results are stratified by gender (M/F). Data are elaborated from the Italian Government (Department of Civil Protection).

Congenital Malformations	Class of Environmental Waste Index (EWI)					
	I	II	III	IV	V	Trend
Total	1	1.09	1.14 *	0.93	1.05	0.99
Nervous System	1	1.22	1.45	0.97	1.83 *	1.08
Neural tube defects	1	1.09	1.22	0.90	1.45	1.02
Cardiovascular	1	1.04	1.16	0.83	0.86	0.95
Oral	1	1.18	0.97	1.19	0.98	1.02
Digestive	1	0.91	0.77	0.86	0.58	0.93
External Genital	1	1.04	1.40	0.83	0.91	0.97
Hypospadias	1	1.16	1.37	0.94	0.94	0.97
Urogenital system	1	1.23	1.26	1.54 *	1.83 *	1.14 *
Skeleton-Muscle	1	1.25	0.90	1.00	1.42	1.01
Limbs	1	0.92	0.99	0.90	0.89	0.98
Chromosomes	1	1.52 *	1.39	0.89	0.97	0.94

* Statistically significant (*p* < 0.05).

The examined studies have provided evidence that levels of dioxins were higher than expected in milk samples produced by mammals bred into the provinces of Naples and Caserta [16,26,27], which strongly suggests that the food chain seems to be somehow compromised in this geographical area and urges to improve controls in local farms in order to verify the health and toxicological status of all animals (including breeding and the fodder used to feed them). The highest level of dioxins was observed in sheep’s milk, as compared to those observed in milk samples from both cattle and buffaloes; this finding could be explained by the fact that a large fraction of sheep are fed by soil, which contains higher levels of dioxins (the permitted limit of dioxins is 10 ng TEQ/Kg of soil) in comparison to those permitted for dried grass (0.75 ng TEQ/Kg of fat). Human contamination by dioxins and dl-PCBs has been found in the large study carried out by Rivezzi *et al*. and by Giovannini *et al*., showing a diffuse environmental contamination and statistically significant association with age the of the sampled women (bioaccumulation effect already taking place), concentration in buffalo milk samples collected in the study area, and the presence of the illegal burning of solid wastes [28,30].

However, dioxins are not the only problem present in these two provinces. In fact, many illegal waste sites have been found to contain dangerous chemicals such as mineral oil, lead, mercury, aluminum residuals, arsenic, and tire residuals; the levels of such products have steadily increased in the last five years (the increase has been estimated at about 30%) [5]. The total environmental infractions for illegal waste disposal recorded by the authorities in Campania during 2006 were 3169 (this number is the highest among all Italian regions), with 1362 interdicted sites [5]. Moreover, ground water samples from the same areas have been found to contain several hazardous chemicals, such as toluene, tricholoroethilen, tetra-cloro-ethilene, and heavy metals [5]. As a consequence, local authorities have forbidden the use of water wells for agricultural use in many sites of the Caserta and Naples provinces. In the same geographical area, SMR for all cause and cause-specific mortality as well as risks for congenital malformations are significantly higher than expected. These data cannot provide evidence of a causal relationship between toxic wastes, dioxins, and health impairments in Campania; actually, mortality for all causes of deaths and overall cancer mortality in the entire region has shown a decreasing trend from 1990 to 2012 (and even if considering data from different provinces, it could be difficult to associate illegal dumping with an increased general effect on mortality).

However, available evidence surely indicates that the dissemination of illegal waste dumping should be mitigated through the improvement of a proper waste management chain, while monitoring of dioxin levels in this area should be improved in order to have an adequate control of the public health [35,36]. In this respect, the Campania region would need to establish a new environmental protection and prevention policy. Such a policy should foresee: (a) the induction of higher levels of differential waste and the start of a recycling project through capillary information and education of people living in this area, especially in the Naples and Caserta provinces; (b) the improvement of the waste selector efficiency; (c) the eradication of illegal burning and waste sites through major control of the legal forces in the territory (improvement of police action); (d) the increase of the monitoring of environmental pollution through screening of biological samples from domestic animals bred into this geographical area (especially sheep); (e) the activation of screening programs on exposed human populations for toxicological chemical detection, including assessment of dioxins in human breast milk samples; and (f) the activation of research centers in Campania specifically devoted to the environment and health.

## 4. Conclusions

Environmental and health data summarized in this paper should be considered an indication to focus on the possible risk of human health in Campania. As made evident by Bifulco [37], the Land of Fires could constitute an ideal open-air laboratory for promising exposomic research, in order to characterize pollution types and sources and to elaborate on measures to eliminate or reduce associated health risks. The approval of prevention interventions, according to an established European model, should pass through a rigorous Health Impact Assessment (HIA), based on scientific evidence and on integration of technical expertise, achieving maximum health benefits and minimizing adverse effects. At the same time, it must be pointed out that at the present—as a consequence of the huge attention paid to its regional, specific situation by the media and population—Campania has activated challenging environmental monitoring campaigns on food chain and soil and water contamination, which have not been implemented in other Italian regions. In addition, a recent program for citizen involvement in monitoring and preventing the illegal activities of burning or dumping has been officially activated by the national government delegates and regional authorities under the Europe for Citizens Program (COHEIRS: Civic Observers for Health and Environment: Initiative of Responsibility and Sustainability), ruled by ALDA European agency in Strasbourg, the International Society Doctors for the Environment (ISDE), and ISBEM research Centre.
